# Electropolymerized Aptasensor for Femtogram‐Level Cytokine Detection

**DOI:** 10.1002/adma.74691

**Published:** 2026-08-19

**Authors:** Jiyao Yu, Renan Colucci, Rachel X. Shi, Qi Lu, Tommaso Marchesi D'Alvise, Ulrike Kraft, Christopher V. Synatschke, Tanja Weil

**Affiliations:** ^1^ Synthesis of Macromolecules Max Planck Institute for Polymer Research Mainz Germany; ^2^ Organic Bioelectronics Research Group Max Planck Institute for Polymer Research Mainz Germany; ^3^ Yale School of Medicine New Haven Connecticut USA

**Keywords:** cytokine sensors, IL‐6, nanofilms, OECT, poly(amino‐ʟ‐tyrosine), SPR

## Abstract

Detecting cytokines at ultralow concentrations is important for the early diagnosis and monitoring of inflammatory and immune disorders, yet remains constrained by the sensitivity and stability of bioelectronic interfaces. Here, we present an electropolymerization strategy for preparing poly(amino‐ʟ‐tyrosine) (pALT) nanofilms as functional biointerfaces for DNA aptamer‐based cytokine recognition. Electropolymerization yields ultrathin, continuous, and ultrasmooth coatings that combine chemical stability with accessible carboxyl functionalities for the direct bioconjugation of amine‐terminated aptamers. Integrated into fiber‐optic surface plasmon resonance and organic electrochemical transistor platforms, the pALT interfaces support interleukin‐6 detection with femtogram‐level sensitivity (100 fg mL^−1^) across five orders of magnitude in dynamic range, among the lower reported detection ranges. By coupling controllable electropolymerization with direct surface biofunctionalization, this approach provides a versatile route to optical and bioelectronics sensing interfaces on electrically conductive substrates.

## Graphical abstract

Electropolymerization of 3‐amino‐ʟ‐tyrosine creates ultrathin, smooth, and chemically robust pALT nanofilms with accessible carboxyl functionalities for direct aptamer immobilization. Integrated with fiber‐optic SPR and OECT platforms, these interfaces enable IL‐6 detection down to 100 fg mL^−^^1^ and provide a versatile route to bioelectronic sensing interfaces.

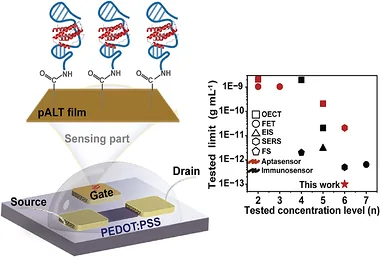

## 1 Introduction

Cytokines orchestrate immune signaling, and minute changes in their concentration within accessible biofluids can foreshadow inflammatory disease [1, 2, 3]. Yet their reliable detection at ultralow levels remains constrained by the sensitivity and stability of bioelectronic interfaces that must translate molecular recognition into reproducible electrical responses [4].

Among these biomarkers, interleukin‐6 (IL‐6) is especially informative: circulating levels in healthy individuals range from 0.2 to 7.8 pg mL^−1^ (approximately 10–370 fM) but rise sharply to ∼1.7 ng mL^−1^ (≈80 pM) in sepsis and ∼8.5 ng mL^−1^ (≈400 pM) in septic shock [5, 6]. This 10^4^–10^5^‐fold span motivates the development of sensing platforms that combine femtogram‐per‐millilitre sensitivity with a broad dynamic range from fg mL^−1^ to ng mL^−1^. Conventional assays such as the enzyme‐linked immunosorbent assay (ELISA) [7] and polymerase chain reaction (PCR) [8] provide benchmark accuracy but require hours of preparation and sophisticated instrumentation, restricting their use in point‐of‐care and wearable formats [9].

At the heart of a biosensor lies the functional interface at which molecular recognition and signal transduction converge [10]. Optimal interfaces should provide accessible recognition sites, limit nonspecific adsorption and morphological defects, and support reproducible signal transduction. Established approaches, such as self‐assembled monolayers (SAMs), nanoparticle constructs, or metal‐organic frameworks [11, 12, 13], achieve molecular precision but often suffer from pinholes, structural heterogeneity, limited stability in physiological environments, and typically require costly materials and multistep fabrication.

Electropolymerization provides a direct and controllable route to conformal coatings on electrically conductive substrates [14]. Electropolymerized films have been generated from monomers such as thiophenes, phenols, anilines, and pyrroles, with polypyrrole representing one of the most established biosensor interfaces owing to its mild aqueous deposition and biocompatibility [15]. Polypyrrole‐based interfaces, however, often require prior monomer functionalization or post‐deposition modification to support bioconjugation, and the resulting film geometry can depend strongly on the deposition conditions [16, 17]. This motivates the development of electropolymerized films that function not only as deposited coatings but also as chemically addressable biointerfaces. 3‐Amino‐ʟ‐tyrosine is attractive because it can be electropolymerized and retains carboxyl‐containing functionalities that permit subsequent bioconjugation.

Here we report bioinspired electropolymerized poly(amino‐ʟ‐tyrosine) (pALT) nanofilms as functional biointerfaces for aptamer‐based cytokine sensing. Electropolymerization produces ultrathin (∼10 nm), ultrasmooth (root‐mean‐square roughness ≈1.5 ± 0.1 nm), and chemically stable coatings with accessible carboxyl groups, enabling direct attachment of amine‐terminated DNA aptamers. When integrated into fiber‐optic surface plasmon resonance (FO‐SPR) and organic electrochemical transistor (OECT) platforms, the pALT interfaces achieve femtogram‐level IL‐6 detection across five orders of magnitude in concentration, among the lower reported detection ranges under buffered conditions. Beyond their analytical performance, the pALT films exemplify how controllable electropolymerization can serve as a practical route to engineering functional biointerfaces for bioelectronic sensing.

## 2 Results and Discussion

### 2.1 Programmable Growth and Structure of pALT Nanofilms

Controlled electropolymerization of 3‐amino‐ʟ‐tyrosine in phosphate buffer provides a straightforward route to chemically characterized films with accessible surface functionalities. Using cyclic voltammetry (CV) in a three‐electrode configuration (Figure 1A), film growth was initiated by sweeping the potential between –0.5 and +1.2 V versus Ag/AgCl. Based on comparison with the electrochemistry of related phenolic and aminophenolic systems, the anodic features at approximately 0.26, 0.72, and 1.02 V (Figure 1B, C) correspond to phenolic oxidation, a higher‐potential aromatic amine‐related oxidative process, and subsequent oxidation and monomer coupling processes, respectively, indicating a cascade of oxidative transformations that propagate the polymer network [14, 18, 19]. The progressive decrease in current density over successive cycles reflects increasing electrode passivation accompanied by continued film growth [20].

**FIGURE 1:**
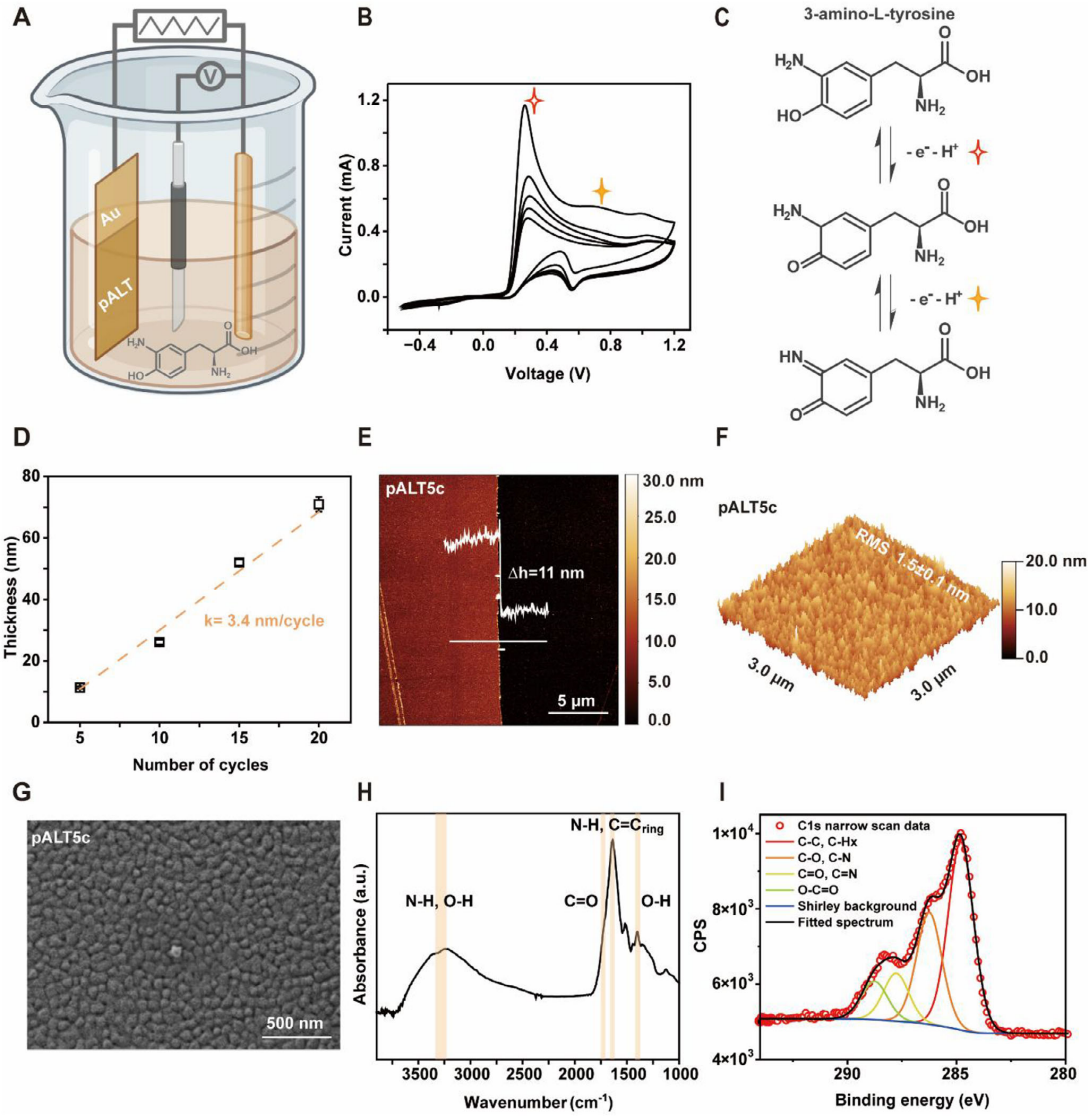
Programmable electropolymerization and characterization of pALT nanofilms. (A) Schematic of 3‐amino‐ʟ‐tyrosine electropolymerization. (B) Representative CV profiles showing anodic oxidation peaks assigned to phenol (red star) and a higher‐potential aromatic‐amine‐related oxidation process (orange star). (C) Conceptual elementary redox steps of the monomer. The overall electropolymerization process is irreversible, and the schematic does not represent a resolved polymer repeating unit. (D) Film thickness versus cycle number. Error bars represent the standard deviations obtained from two to four independently prepared samples, with at least three measurements at different positions on each sample. (E,F) AFM topography and 3D height map of pALT5c, with an RMS roughness of approximately 1.5 nm. (G) SEM image of pALT5c. (H) FT‐IR spectrum showing aromatic, nitrogen‐containing, and carboxyl‐containing features. (I) Representative high‐resolution C1s XPS and its deconvolution into four chemically plausible contributions.

The electropolymerization process enables nanometre‐scale control over film thickness and geometry. Films grown for five CV cycles reached an average thickness of approximately 11 nm, and the fitted thickness increased linearly at approximately 3.4 nm per cycle over the investigated range (Figure 1D). Atomic‐force microscopy (AFM) revealed ultrasmooth topographies with root‐mean‐square (RMS) roughness of 1.5 ± 0.1 nm for both five‐ and ten‐cycle films (Figure 1E, F and Figure S1). At higher cycle numbers, characteristic O‐ and C‐shaped surface motifs emerged (Figures S1 and S2), likely arising from diffusion‐limited self‐oxidation of accumulated monomer at the electrode interface [21, 22]. Hence, films produced after five cycles (hereafter denoted pALT5c) were selected for further investigation, as they provided a continuous and reproducible ultrathin pALT interface while minimizing deposition time and secondary surface heterogeneity.

Spectroscopic analysis provided information on the chemical structure of pALT. Fourier‐transform infrared (FT‐IR) spectrum displayed a prominent band centred at 1636 cm^−1^, assigned primarily to overlapping aromatic ring stretching and N–H deformation modes, with possible contributions from conjugated C═N‐containing structures associated with the formation of indole or phenoxazine motifs (Figure 1H and Figures S3 and S4) [18, 23, 24, 25, 26]. The broad O–H stretching vibration at 3255 cm^−1^ and the C═O band at 1702 cm^−1^ are consistent with carboxylic acid‐containing environments [20]. High‐resolution x‐ray photoelectron spectroscopy (XPS; Figure 1I; Figure S5 and Table S1) revealed a O─C═O component at approximately 288.8 eV accounted for 10.8 ± 0.5% of the C1s envelope across three independently prepared samples, comparable to that of a carboxyl‐terminated 11‐mercaptoundecanoic acid SAM measured under the same conditions (7.7 ± 1.1%; Figure S6), supporting the presence of abundant carboxyl‐containing functionalities for amine‐terminated aptamer immobilization.

The resulting pALT nanofilms exhibited chemical and mechanical robustness under the tested conditions. At the spatial resolution of the SEM measurements, the films retained continuous and crack‐free morphologies after ten days of incubation across pH 2–10 and in various organic solvents (Figure 2A). XPS analysis confirmed persistence of the organic pALT layer, with limited interfacial reorganization under harsher media (Figure 2B, C and Figure S7). Strain‐induced elastic buckling instability for mechanical measurements (SIEBIMM; Figure 2D) yielded the crosslinked pALT nanofilms (via EDC/NHS chemistry), an average Young's modulus of 1.3 ± 0.2 GPa, slightly higher than 0.9 ± 0.2 GPa measured for the poly(3,4‐ethylenedioxythiophene) polystyrene sulfonate (PEDOT:PSS) layer [27]. In addition, the transferred films supported on flexible polycarbonate membranes withstood large deformations without visible cracking (Figure 2E). This combination of chemical stability, mechanical resilience, and surface smoothness establishes pALT5c as a robust interfacial material for subsequent aptamer functionalization and device integration.

**FIGURE 2:**
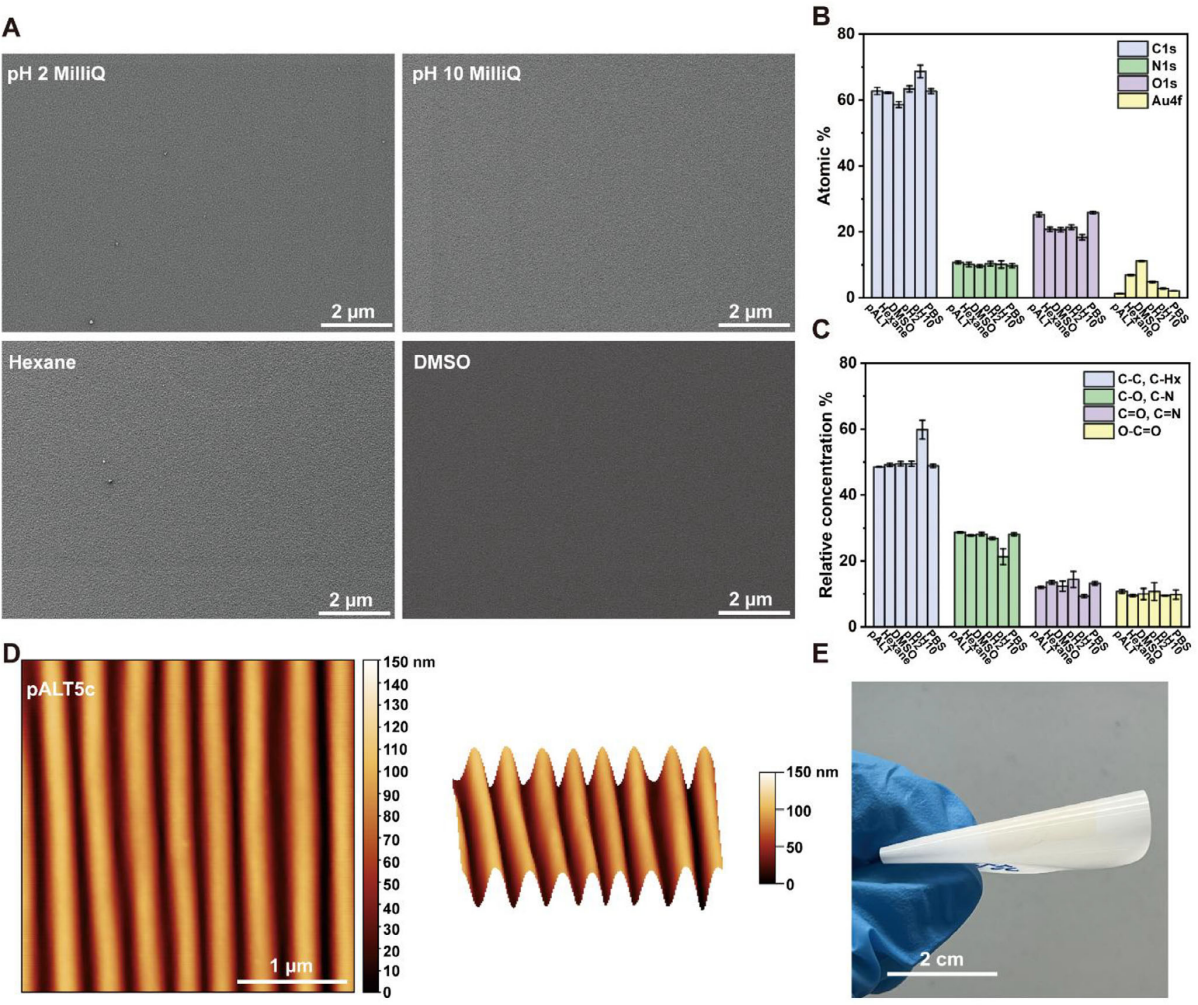
Chemical and mechanical stability of pALT nanofilms. (A) SEM images showing that the films remain continuous and crack‐free after incubation in different media. Milli‐Q water at pH 2 and pH 10 were selected to test stability under acidic and basic conditions, respectively; hexane was used as a nonpolar organic solvent, and DMSO as a strongly polar aprotic solvent. (B) Survey XPS‐derived elemental composition of the incubated pALT films. C1s, N1s, and O1s signals were retained under all tested conditions, confirming persistence of the organic pALT layer. The increased Au4f signal observed after DMSO, hexane, and pH 2 incubation indicates increased visibility of the underlying Au substrate, consistent with limited interfacial reorganization and/or partial removal of weakly bound oligomeric species. (C) Relative C1s component distribution obtained from high‐resolution C1s fitting. The largely preserved component distribution supports retention of the main pALT chemical structure, while the pH 10‐treated sample showed increased C–C/C–Hx and decreased C–O/C–N contributions, suggesting alkaline‐induced reorganization or further coupling/crosslinking. (D) AFM image and the 3D image of pALT5c nanofilms transferred to polydimethylsiloxane (PDMS) substrates. The corresponding Young's modulus of pALT5c is 1.3 ± 0.2 GPa, as determined from three independent measurements. (E) Photograph of the flexible pALT5c nanofilms supported by a polycarbonate membrane.

### 2.2 Preparation and Characterization of the Aptamer‐Containing pALT Interface

Molecular recognition is the basis of selective biological responses, where precise binding events underpin immune defence and cellular communication [28]. To emulate this selectivity within synthetic interfaces, we functionalized pALT nanofilms with DNA aptamers, creating a biomimetic sensing platform for ultrasensitive cytokine detection.

DNA aptamers, synthetic single‐stranded oligonucleotides, offer high specificity for their target analytes [29, 30] and can regenerate their binding conformation after complex dissociation [31]. The abundant surface carboxyl groups on pALT enable direct covalent coupling of amine‐terminated aptamers through standard 1‐ethyl‐3‐(3‐dimethylaminopropyl)carbodiimide/*N*‐hydroxysuccinimide (EDC/NHS) activation chemistry (Figure 3A). This one‐step conjugation establishes a functionally active biorecognition layer without requiring additional linker molecules.

**FIGURE 3:**
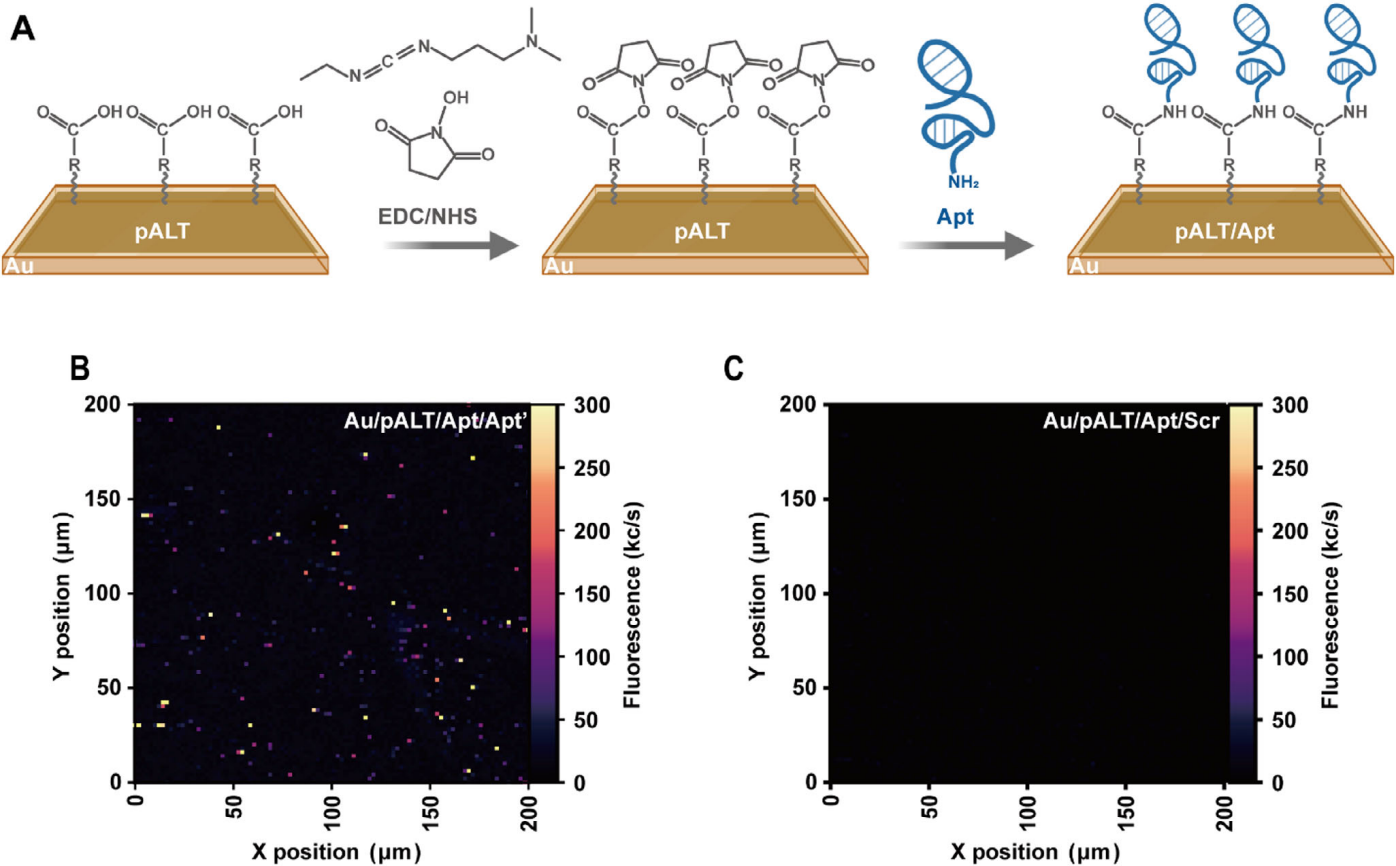
Aptamer functionalization and characterization. (A) Schematic of aptamer immobilization on pALT‐coated gold substrates via EDC/NHS coupling. Confocal fluorescence micrographs of aptamer‐functionalized pALT nanofilms following incubation with: (B) Cy3‐labeled complementary strand (Apt′) and (C) Cy3‐labeled scrambled control sequence (Scr). Bright fluorescence appears exclusively for Apt′ hybridization, confirming specific aptamer immobilization.

The success and fidelity of the immobilization process were verified by confocal fluorescence microscopy. As the aptamer (Apt) itself is nonfluorescent, hybridization assays were performed using a Cyanine‐3 (Cy3)‐labeled complementary probe (Apt’) and a Cy3‐labeled scrambled control sequence (Scr). Strong fluorescence was observed only when the complementary probe hybridized to aptamers immobilized on the pALT film (Figure 3B), whereas neither the nonfunctionalized surface (Figure S8) nor the scrambled control (Figure 3C) produced detectable emission. These results confirm (i) efficient immobilization of the aptamer, (ii) preservation of its secondary structure and binding capacity, and (iii) minimal nonspecific adsorption. In addition, low‐magnification wide‐field fluorescence imaging (Figure S9) showed Cy3 signal over the sample area, supporting macroscopic coverage of the functionalized surface. A Cy3‐labeled complementary‐strand depletion assay further estimated the hybridization‐accessible aptamer coverage to be 2.69 ± 1.01 pmol mm^−2^, supporting sufficient receptor availability for IL‐6 sensing (Figure S10). Supporting FT‐IR and SEM analyses (Figures S11 and S12) further corroborated the formation of a biofunctionalized layer.

Together, these findings demonstrate that the accessible carboxyl sites within pALT films enable quantitative and structurally preserved aptamer grafting, establishing a chemically defined, continuous, and crack‐free biointerface for subsequent cytokine sensing.

### 2.3 pALT Nanofilm‐Based Cytokine Aptasensor

Having established a robust and selective biointerface, we next evaluated the aptamer‐functionalized pALT films as transduction layers for cytokine sensing. The aptamer‐functionalized nanofilms were integrated into fiber‐optic (FO) SPR and OECT platforms to monitor IL‐6 binding in real time (Figure 4). These complementary configurations probe distinct aspects of the interfacial response: FO‐SPR captures refractive‐index changes associated with biomolecular adsorption, whereas OECTs translate charge redistribution at the gate electrode into amplified electronic signals [32, 33].

**FIGURE 4:**
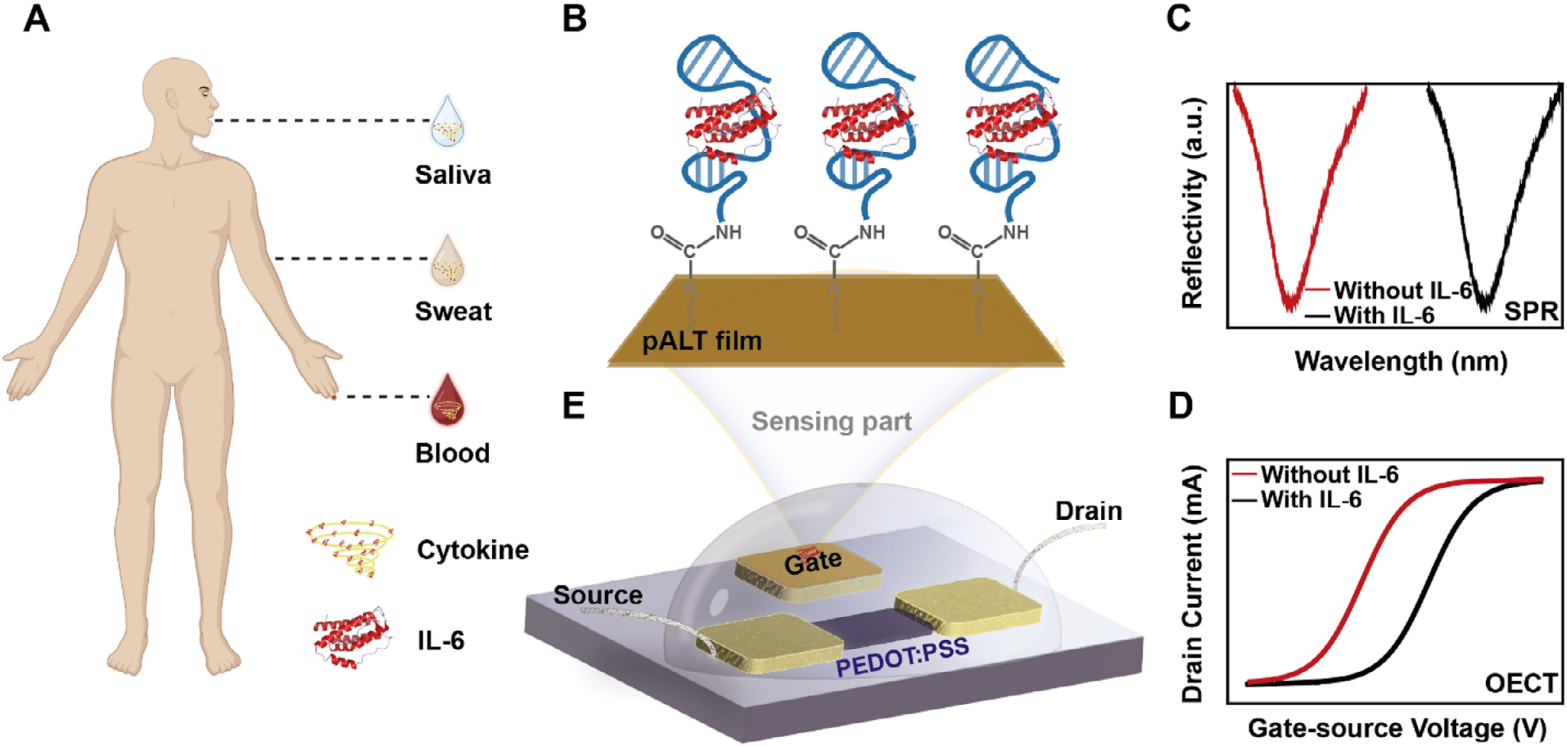
Concept and operation of the pALT‐based cytokine aptasensor. (A) Cytokines such as IL‐6 are present in diverse biofluids, including blood, saliva, and sweat. (B) Schematic of IL‐6 binding to surface‐immobilized aptamers on pALT nanofilms. The recognition event modulates the interfacial environment, giving rise to (C) a wavelength shift in FO‐SPR or the current modulation of OECTs (D) with functionalized pALT‐gate electrodes (E).

In both architectures, selective recognition arises from aptamer‐cytokine binding at the pALT surface (Figure 4B). When IL‐6 in biofluids (e.g., saliva, sweat, or blood serum, as conceptually illustrated in Figure 4A) interacts with the immobilized aptamers, negatively charged protein molecules approach the surface, altering either the local optical environment (FO‐SPR) or the surface potential (OECT). These perturbations manifest as a wavelength shift in FO‐SPR spectra (Figure 4C) [34] or as a gate‐voltage shift in the OECT transfer curve [35] (Figure 4D).

This dual‐platform integration highlights the versatility of the pALT interface: the same molecular film can support both optical and electronic transduction, enabling cross‐validation of recognition events through independent physical observables. Furthermore, the compatibility of the electropolymerization process with metallic and conductive polymeric substrates underscores the scalability of the approach toward multiplexed or flexible diagnostic architectures.

### 2.4 Biosensing Performance and Signal Transduction

#### 2.4.1 Optical Transduction via FO‐SPR

FO‐SPR measurements provide a direct optical probe of interfacial molecular interactions. The custom‐designed setup (Figure 5A) consists of a gold‐coated optical fiber that supports surface‐plasmon excitation and back‐reflected spectral analysis in real time [14, 23]. During electropolymerization, the resonance wavelength progressively red‐shifted, displaying five discrete increments that correspond to the five successive voltammetric cycles involved in film formation (Figure S13). Subsequent EDC/NHS activation, aptamer immobilization, and quenching induced an additional 13 nm shift, confirming further film crosslinking and attachment of the aptamer layer (Figure 5B).

**FIGURE 5:**
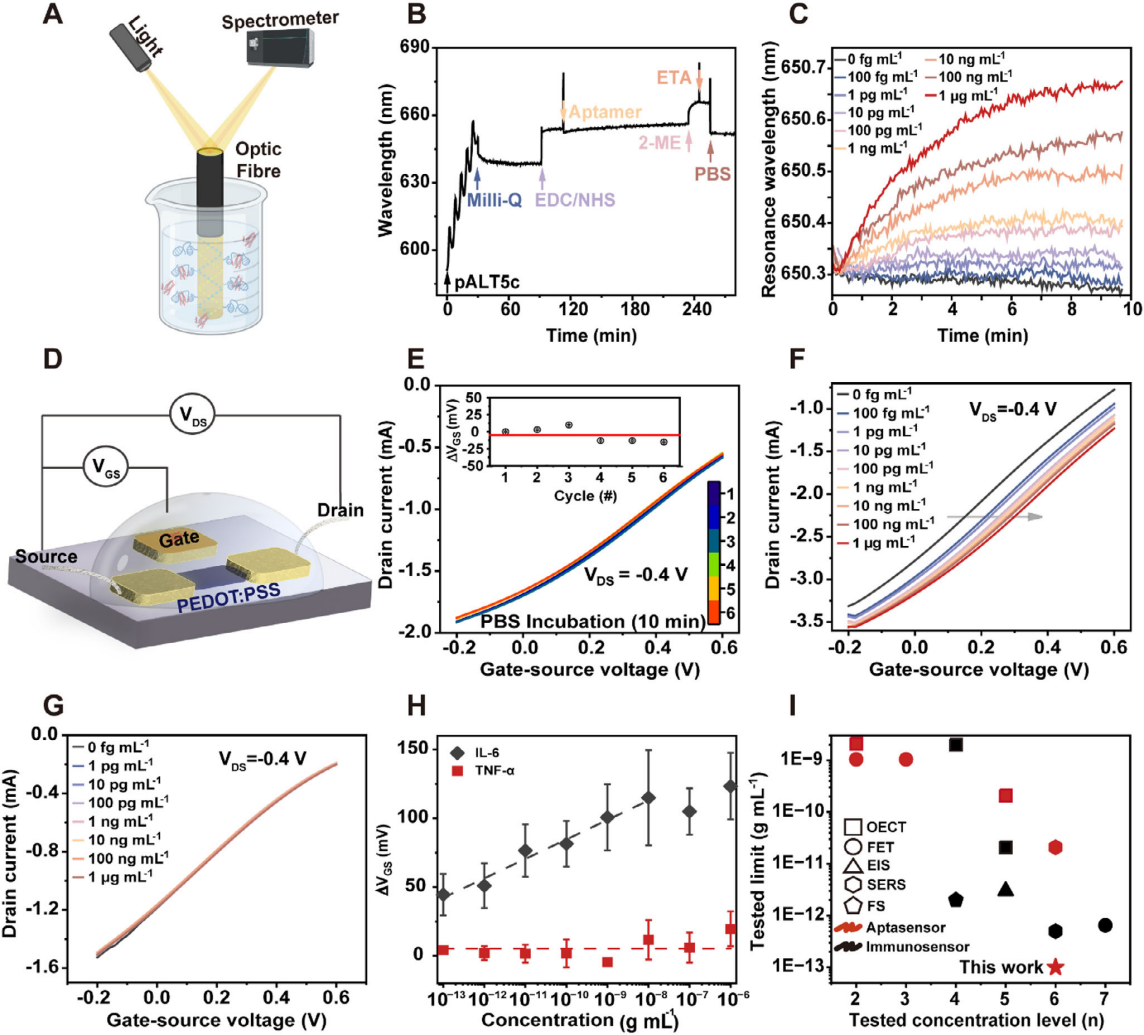
Biosensing performance of the pALT‐based cytokine aptasensor. (A) Schematic of the FO‐SPR setup. (B) Real‐time resonance shifts during electropolymerization and aptamer functionalization. (C) FO‐SPR response to IL‐6 at different concentrations. Data were extracted from one continuous measurement on a single sensor and baseline‐normalized for comparison. The full trace and independent repeats are shown in Figure S14A, B. (D) Planar OECT configuration with pALT‐coated Au gate. (E) Stability of OECTs over six consecutive incubation cycles in PBS. Inset: gate‐voltage shift over six consecutive cycles, calculated from the current value at *V_GS_* = 0.2 V with *V_DS_* fixed at –0.4 V. (F, G) Transfer‐curve shifts upon exposure to IL‐6 and TNF‐α. (H) Analytical calibration of Δ*V_GS_* versus cytokine concentration. The error bars were obtained from the standard deviation of five devices for IL‐6 and four devices for TNF‐α. The black dashed line represents the linear detection range for IL‐6, and the red dashed line represents the average of the variation of the gate voltage for TNF‐α. (I) Comparison of tested lower concentration limits and the reported linear range with state‐of‐the‐art IL‐6 biosensors.

Next, the response of the proposed aptasensor was investigated at different concentrations of IL‐6 (Figure 5C and Figure S14). When exposed to IL‐6 solutions spanning 100 fg mL^−1^ to 1 µg mL^−1^, the resonance wavelength increased proportionally to concentration. Moreover, the measurements in human serum albumin (HSA) solution produced no discernible signal variation, indicating negligible nonspecific adsorption (Figure S15). Control FO tips lacking aptamers showed no measurable change (Figure S16), verifying that the signal originates from specific IL‐6 binding. However, at low concentrations (≤1 ng mL^−1^), the observed shifts approached the spectrometer's resolution limit (∼0.1 nm, Figure 5C), motivating the use of OECTs to amplify the interfacial signal electrically.

#### 2.4.2 Electrochemical Transduction via OECTs

The OECT configuration (Figure 5D) features a planar configuration [36] with gold source and drain contacts and a gate electrode fabricated via photolithography [37, 38]. The device exploits volumetric doping and de‐doping within a PEDOT:PSS channel (thickness ≈ 40–60 nm; *W* × *L* = 1 × 0.3 mm^2^, see Figure S17 and Table S2) gated through an electrolyte by a pALT‐coated Au electrode [39], enabling low‐voltage operation with intrinsic signal amplification, thus outperforming conventional electrochemical biosensors [33, 40, 41, 42] and enabling low limits of detection [35].

First, the electrical characteristics of the pristine OECT devices were evaluated to ensure their proper operation. The output and transfer characteristics measured in PBS exhibited the characteristic depletion‐mode behavior (Figure S18) [36]. Upon applying a positive gate voltage, cations from the electrolyte migrate into the channel, de‐doping the PEDOT:PSS and reducing the drain current [43]. The transconductance (*gm*), determined as the first derivative of the transfer curve, reached peak values of a few‐mS, with the maximum occurring at approximately *V_GS_* = 0.2 V and *V_DS_* = −0.4 V, consistent with the literature [43, 44, 45]. Furthermore, the device maintained stability over multiple PBS incubation cycles (10 min each, Figure 5E).

After validating the operation of the pristine OECTs, we examined the sensing response of the proposed aptasensor (Figure 5F and Figure S19). Upon IL‐6 binding, the transfer curves shifted systematically toward higher (more positive) gate voltages, consistent with previous reports [46, 47]. This behavior arises from interfacial charge compensation: IL‐6 carries a net negative charge at physiological pH [48], and its association with the aptamer decreases the effective gate potential (Figures S21A and S22), requiring a larger bias to sustain the same drain current. A more comprehensive mechanism analysis is provided in the Supporting Information. In contrast, introducing the non‐target cytokine tumour necrosis factor‐α (TNF‐α) with a similar molecular weight to IL‐6 induced negligible and non‐monotonic shifts (Figure 5G), confirming specificity.

Next, the sensing response was quantified as the shift in gate voltage (Δ*V_GS_*), extracted from the current value at *V_GS_* = 0.2 V (*V_GS_* with the maximum transconductance, Figure S18B) with *C_prot_* = 0 as the reference and plotted against cytokine concentrations in Figure 5H. This metric directly captures the binding‐induced change in the surface potential underlying the sensing response and enables comparison with the independently measured potential shifts. For IL‐6 cytokine detection, the gate‐voltage shift (Δ*V_GS_*) exhibited a linear dependence on the logarithm of the IL‐6 concentration from 100 fg mL^−1^ to 10 ng mL^−1^ (Figure 5H), beyond which only minimal changes were observed, indicating signal saturation from 10 ng mL^−1^ to 1 µg mL^−1^. In contrast, for the nonspecific TNF‐α cytokine, the response remained negligible across the entire tested concentration range, which is in good agreement with the Kelvin probe results (Figure S21B). The limit of detection (LOD), determined following International Union of Pure and Applied Chemistry (IUPAC) criteria (Figure S23) [49], was 10 fg mL^−1^, corresponding to roughly 0.48 fM. This performance surpasses that of state‐of‐the‐art IL‐6 sensors based on OECTs [12, 46, 47, 50], field‐effect transistors (FET) [51, 52, 53], electrochemical impedimetric biosensors (EIS) [54], surface‐enhanced Raman spectroscopy (SERS) biosensors [30, 55], and surface plasmon‐enhanced fluorescence spectroscopy biosensors (FS) [56] (Figure 5I; Table S3).

The combined FO‐SPR/OECT results demonstrate that the electrochemically grown pALT interface enables efficient translation of selective aptamer‐based molecular recognition into amplified optical and electrical signals, bridging the molecular and device scales of bioelectronic transduction. To assess storage stability, the sensing performance was further evaluated using both FO‐SPR and OECT platforms after 3 and 7 days. The results showed that the pALT‐based interface maintained IL‐6 sensing capability, although prolonged storage reduced the magnitude of the sensing signal (Figure S24).

## 3 Conclusion

Advances in nanomaterials have enabled diverse strategies for engineering biosensor interfaces, including SAMs, nanoparticle constructs, metal‐organic frameworks, and carbon‐based scaffolds. SAMs provide high molecular‐level control, especially on well‐defined gold surfaces, but their performance can be affected by substrate crystallinity, defect formation, and surface reorganization in physiological media [57]. Here we show that electropolymerized pALT nanofilms provide a minimalist yet powerful alternative, combining polymer‐like processability with the robustness of an inorganic coating.

The pALT films exhibit chemical and mechanical stability, remaining continuous across pH 2–10 and in diverse solvents, while maintaining ultrasmooth morphologies (RMS ≈1.5 ± 0.1 nm). The accessibility of carboxylic acid groups allows reproducible aptamer immobilization through standard EDC/NHS coupling. Although the precise molecular architecture of pALT is still under investigation, spectroscopic signatures and literature precedents suggest the formation of phenoxazine‐like and indole‐type motifs. Redox‐probe EIS and cyclic voltammetry (Figure S25) indicated that pALT forms a less charge‐transfer‐blocking interface than carboxyl‐terminated 11‐MUA SAMs. These attributes collectively underpin the film's ability to mediate stable and efficient signal transduction.

When integrated into FO‐SPR and OECT architectures, the consistent performance across optical and electrochemical transduction modalities underscores the versatility of the electrochemically grown interface. Because the detection relies solely on aptamer‐target recognition, the platform can be readily reconfigured for other analytes by exchanging the aptamer sequence, without altering the underlying transduction layer. The mechanical flexibility and substrate compatibility of pALT films further enable integration into wearable, low‐power electronic devices, paving the way for continuous, real‐time monitoring of inflammatory biomarkers. Moreover, the simplicity and scalability of the electropolymerization process make it adaptable to diverse conductive materials and device geometries, offering a practical path toward industrial‐scale implementation.

In summary, this work establishes bioinspired electropolymerized pALT nanofilms as functional biointerfaces for ultrasensitive biosensing. Integrated within optical (FO‐SPR) and electrochemical (OECT) architectures, these films achieve femtogram‐level IL‐6 detection across five orders of magnitude in concentration. Beyond demonstrating record sensitivity, this approach introduces a general biofunctional layer design principle, linking controlled redox polymerization to selective molecular recognition, offering a versatile platform for next‐generation diagnostic and bioelectronic technologies.

## 4 Experimental Section

### 4.1 Fabrication of pALT Nanofilms

pALT nanofilms were deposited by electropolymerization using a Metrohm Autolab PGSTAT204 potentiostat in a standard three‐electrode configuration. A gold wire served as the counter electrode, an Ag/AgCl (3 M KCl) electrode as the reference, and the working electrode was either a gold‐coated glass slide (100 nm Au), a gold‐coated optical fiber tip (35 nm Au), or an OECT gold gate electrode (100 nm Au).

Electropolymerization was performed in a 1 mg mL^−1^ 3‐amino‐ʟ‐tyrosine solution prepared in 100 mM phosphate buffer (pH 7.0). The potential was cycled between –0.5 V and +1.2 V at a scan rate of 0.01 V s^−1^ for the desired number of cycles. After polymerization, the substrates were rinsed thoroughly with Milli‐Q water to remove residual oligomers and dried under nitrogen before further use.

### 4.2 Immobilization of Aptamers

Lyophilized aptamers were reconstituted to 100 µM in nuclease‐free water, aliquoted (20 µL), and stored at –20°C. Before immobilization, aptamers were thermally conditioned by denaturation at 95°C for 5 min, rapid cooling on ice for 15 min, and equilibration at room temperature for 5 min to ensure proper secondary structure formation.

Gold‐coated substrates bearing pALT nanofilms were incubated in an aqueous solution of EDC/NHS (200 mM/50 mM) for 20 min to activate surface carboxyl groups. Subsequently, a 10 µM amine‐terminated aptamer solution was applied for 2 h at room temperature to form covalent amide bonds. Residual EDC was quenched using 20 mM 2‐mercaptoethanol (2‐ME) in 100 mM 2‐(N‐morpholino)ethanesulfonic acid (MES) buffer (pH 6) for 10 min, followed by blocking unreacted carboxyl sites with 500 mM ethanolamine (ETA) in PBS (pH 7.4) for 10 min. Finally, the substrates were rinsed three times with PBS (pH 7.4) for 30 min each to remove unbound species and either measured immediately or stored at 4°C until use.

### 4.3 Preparation of Cytokine Samples

Recombinant IL‐6 and TNF‐α were reconstituted to 100 µg mL^−1^ in PBS, aliquoted (18 µL), and stored at –20°C. Working solutions spanning 100 fg mL^−1^‐1 µg mL^−1^ were prepared by serial dilution in PBS. Dilutions were prepared using sterile polypropylene tubes. Independent dilution series were prepared for each experimental repeat. Adsorption losses at femtogram‐per‐millilitre concentrations were not independently quantified; the reported concentrations therefore represent nominal prepared concentrations.

### 4.4 Cytokine Detection

All biosensing measurements were conducted in PBS using either the FO‐SPR or OECT platform.

FO‐SPR measurements. Optical fibers were equilibrated in PBS until a stable baseline was obtained and were then sequentially exposed to increasing IL‐6 concentrations for 10 min per step. HSA and aptamer‐free pALT controls were examined using the procedures described in Figures S15 and S16. The back‐reflected spectra were recorded continuously, and wavelength shifts were extracted in real time.

OECT measurements. Output and transfer characteristics were first recorded in PBS until a stable baseline was obtained. The pALT‐coated gate electrode was then sequentially exposed to increasing cytokine concentrations. At each concentration, 50 µL of cytokine solution was applied to the pALT‐coated gate electrode for 10 min, followed by rinsing with PBS to remove nonspecific adsorbates before OECT measurement. The device channel and gate were then covered with PBS for electrical readout. This incubation‐rinsing‐measurement cycle was repeated for each subsequent higher concentration. The drain‐source voltage (*V_DS_*) was fixed at –0.4 V, while the gate‐source voltage (*V_GS_*) was swept from –0.2 V to +0.6 V. The gate‐voltage shift (Δ*V_GS_*) was calculated from the current at *V_GS_* = 0.2 V using the signal at zero protein concentration (*C_prot_* = 0) as reference. Five independently fabricated devices were used for IL‐6 detection and four for TNF‐α detection.

### 4.5 Limit of Detection (LOD)

The limit of detection was determined according to IUPAC guidelines [49] using the following equation:

(1) $$LOD=10^{\frac{\left[{\left(\Delta V_{GS}\right)}_{blank}+3\sigma \right]-Intercept}{Slope}}$$

where (Δ*V_GS_*)_blank_ is the average shift in the gate voltage observed upon incubation of the aptasensor in PBS (in the absence of the analyte, Figure S23), σ is the respective standard deviation, and the factor of 3 ensures a confidence level of 99.86%. The intercept and slope are obtained from the linear regression of the analytical calibration curve.

### 4.6 Additional Characterization

Detailed procedures for nanofilm characterization, and fabrication and calibration of OECT and FO‐SPR devices, as well as the detection mechanism, are provided in the Supporting Information.

## Author Contributions

J.Y., R.C., R.X.S., and T.M.D. conceived and designed the experiments. R.X.S. and T.M.D. conducted preliminary studies. J.Y. fabricated and characterized pALT nanofilms, performed SPR, and performed data analysis. R.C. fabricated OECTs and carried out EIS and Kelvin‐probe experiments. Q.L. performed confocal fluorescence microscopy. U.K. contributed to project conceptualization, supervised the project with respect to OECTs, and secured funding. C.V.S. contributed to project conceptualization and supervised the project. T.W. contributed to overall project conceptualization and supervision and secured funding. J.Y. and R.C. wrote the initial manuscript. All authors contributed to data interpretation, manuscript revision, and approved the final version.

## Conflicts of Interest

The authors declare no conflicts of interest.

## Supporting information




**Supporting File**: adma74691‐sup‐0001‐SuppMat.docx.

## Data Availability

All data supporting the findings of this study are available within the Supporting Information or from the corresponding authors upon reasonable request.
