# Supplementary material for: Electropolymerized Aptasensor for Femtogram‐Level Cytokine Detection
Source: Adv Mater. 2026 Aug 19;38(58):e74691. doi: 10.1002/adma.74691 (PMC13674618; doi:10.1002/adma.74691)
Supplement: Supplementary file 1 [file ADMA-38-e74691-s001.docx]

Supporting Information

Electropolymerized Aptasensor for Femtogram-level Cytokine Detection

Jiyao Yu^1^†, Renan Colucci^2^†, Rachel X. Shi^3^, Qi Lu^1^, Tommaso Marchesi D'Alvise^1^, Ulrike Kraft^2^*, Christopher V. Synatschke^1^*, Tanja Weil^1^*

1 Synthesis of Macromolecules, Max Planck Institute for Polymer Research, Ackermannweg 10, 55128 Mainz, Germany

2 Organic Bioelectronics Research Group, Max Planck Institute for Polymer Research, Ackermannweg 10, 55128 Mainz, Germany

3 Yale School of Medicine, 333 Cedar St, New Haven, CT 06510, USA

† These authors contributed equally to the work

**Table of Contents**

[Supporting Methods	1](#_Toc234403604)

[1. Materials and chemicals	1](#_Toc234403605)

[2. FO-SPR and OECT devices	1](#_Toc234403606)

[2.1 Preparation of fibre optic (FO) probes	1](#_Toc234403607)

[2.2 Fibre optic surface plasmon resonance (FO-SPR)	1](#_Toc234403608)

[2.3 Fabrication of organic electrochemical transistors (OECTs)	2](#_Toc234403609)

[2.4 OECT electrical measurements	3](#_Toc234403610)

[3. Characterization Methods	3](#_Toc234403611)

[3.1 Atomic force microscopy (AFM)	3](#_Toc234403612)

[3.2 Scanning electron microscopy (SEM)	3](#_Toc234403613)

[3.3 X-ray photoelectron spectroscopy (XPS)	3](#_Toc234403614)

[3.4 Fourier transform infrared spectroscopy (FT-IR)	3](#_Toc234403615)

[3.5 Kelvin probe measurements (KP)	4](#_Toc234403616)

[3.6 Confocal microscopy measurements:	4](#_Toc234403617)

[3.7 Electrochemical impedance spectroscopy (EIS) on OECT	4](#_Toc234403618)

[3.8 Strain-induced elastic buckling instability for mechanical measurements (SIEBIMM)	4](#_Toc234403619)

[3.9 Semi-quantitative estimation of hybridization-accessible aptamer coverage	5](#_Toc234403616)

[3.10 Low-magnification wide-field fluorescence imaging	5](#_Toc234403617)

[3.11 Sensor stability tests after storage	5](#_Toc234403618)

[3.12 Redox-probe electrochemical impedance spectroscopy (EIS) and cyclic voltammetry (CV) on Au substrates	6](#_Toc234403619)

[Supporting Text	7](#_Toc234403620)

[Discussion on the detection mechanism	7](#_Toc234403621)

[Supporting Figures	9](#_Toc234403622)

[Figure S1. AFM height images of pALT films	9](#_Toc234403623)

[Figure S2. SEM images of pALT films	10](#_Toc234403624)

[Figure S3. Proposed structures for the electropolymerized 3-amino-ʟ-tyrosine analogues	11](#_Toc234403625)

[Figure S4. FT-IR spectrum of 3-amino-ʟ-tyrosine	12](#_Toc234403626)

[Figure S5. XPS spectra of pALT film	13](#_Toc234403627)

[Figure S6. XPS spectra of 11-mercaptoundecanoic acid (11-MUA)	14](#_Toc234403628)

[Figure S7. XPS spectra of pALT films after ten days of incubation in different media	16](#_Toc234403629)

[Figure S8. Confocal microscopy of pALT films	17](#_Toc234403630)

[Figure S9. Low-magnification wide-field fluorescence image of pALT films	18](#_Toc234403631)

[Figure S10. Semi-quantitative estimation of hybridization-accessible aptamer coverage	19](#_Toc234403632)

[Figure S11. FTIR spectra of pALT and aptamer functionalized films	20](#_Toc234403633)

[Figure S12. SEM image of aptamer functionalized pALT films	21](#_Toc234403634)

[Figure S13. Real-time resonance and current traces for pALT5c electropolymerization	22](#_Toc234403635)

[Figure S14. SPR measurements for aptasensor in response to IL-6 concentrations	23](#_Toc234403636)

[Figure S15. SPR measurements (control with HSA)	24](#_Toc234403636)

[Figure S16. SPR measurements (negative control)	25](#_Toc234403637)

[Figure S17. Thickness of PEDOT:PSS.	26](#_Toc234403638)

[Figure S18. OECT electrical measurements	27](#_Toc234403639)

[Figure S19. Cyclic voltammetry profiles for a pALT5c electropolymerization procedure	28](#_Toc234403640)

[Figure S20. EIS measurements	29](#_Toc234403641)

[Figure S21. Kelvin probe measurements	30](#_Toc234403642)

[Figure S22. Open-circuit potential measurements	31](#_Toc234403643)

[Figure S23. Limit of detection of aptasensor	32](#_Toc234403644)

[Figure S24. Sensing stability of aptamer-functionalized pALT sensors	33](#_Toc234403645)

[Figure S25. Comparison of interfacial charge-transfer properties of pALT5c- and 11-MUA SAM-modified Au electrodes	34](#_Toc234403646)

[Supporting Tables	35](#_Toc234403647)

[Table S1.	35](#_Toc234403648)

[Table S2.	36](#_Toc234403649)

[Table S3.	37](#_Toc234403650)

[References	39](#_Toc234403651)

# Supporting Methods

## 1. Materials and chemicals

Gold-coated (1000 Å) microscope slide, 3-amino-ʟ-tyrosine dihydrochloride monohydrate (98%), potassium phosphate monobasic (99%), sodium phosphate dibasic (99%), sodium chloride, potassium chloride, 11-Mercaptoundecanoic acid (11-MUA), 2-mercaptoethanol (2-ME), ethanolamine, N-(3-(dimethylamino)propyl)-N’-ethyl-carbodiimide (EDC), N-hydroxysuccinimide (NHS), 2-(N-morpholino)ethanesulfonic acid (MES), Dimethyl sulfoxide (DMSO), hexane, poly(vinyl alcohol) (PVA, 80% hydrolyzed, M_W_ of 8000-10000), human serum type AB, human serum albumin (HSA), Recombinant Human TNF-α, aptamers including IL-6 specific aptamer (Apt, 5’-[AminoC6]CTTCCAACGCTCGTATTGTCAGTCTTTAGT-3’), Cy3-labeled complementary sequence (Apt’, 5’-[Cyanine3]ATAAATACGAGCGTTGGAAGT-3’), Cy3-labeled scrambled sequence (Scr, 5’-[Cyanine3]GATCAGATAGTAGAGATAGTC-3’) were purchased from Sigma-Aldrich. Potassium ferri/ferrocyanide (99%) was purchased from Acros Organics. Recombinant Human IL-6 was purchased from Thermo Fisher Scientific. SYLGARD™ 184 silicone elastomer base and curing agent were purchased from Dow.

## 2. FO-SPR and OECT devices

### 2.1 Preparation of fibre optic (FO) probes

FO probes were prepared according to previously reported procedures^1-3^. FO probes (7.5 cm) were cut from Technology Enhanced Clad Silica (TECS-Clad) Multimode Optical Fibre Cable (Thorlabs, core diameter: 1 mm, numerical aperture: 0.39). A 1 cm section of the outer plastic jacket was mechanically stripped and then immersed in acetone for 4 h to dissolve the inner polymer cladding. The FO tips were sequentially polished using 3 µm, 1 µm, 0.3 µm, and 0.1 µm grit sandpaper to obtain a flat and smooth cross-section. FO tips were then immersed in acetone for 1 h, rinsed with Milli-Q water, and dried with nitrogen.

Regarding the gold sputtering, the fibres were vertically mounted onto the rotating stage of an EM ACE600 sputter coater (Leica Microsystems), and a 0.5 nm titanium adhesion layer, followed by a 35 nm gold layer with a 0.12 nm s^-1^ sputter rate, was deposited onto the exposed FO tips. The opposite fibre ends were similarly stripped by 1 cm, immersed in acetone, and polished to enable efficient optical coupling. To ensure reliable electrical contact between the gold-coated jacketed section and the optically sensing tip, the transitional region was coated with conductive silver paint (Ag-paint, RS Components, Ltc), air-dried for 2 h, and then encapsulated with liquid electrical insulator (Performix Liquid Tape, Plasti Dip Europe GmbH), followed by an additional drying step. Finally, to enhance the stability of the sputtered gold during subsequent electropolymerization procedures, the fibres were thermally annealed at 150 °C for 4 h.

### 2.2 Fibre optic surface plasmon resonance (FO-SPR)

FO-SPR measurements were performed following established procedures^1, 2^. Briefly, the gold-coated FO probes were cleaned using argon plasma for 10 min at 6 mbar. Each FO probe was connected to a Y-shaped optical splitter via a bare fibre terminator (Thorlabs). Polychromatic light was delivered through the splitter to the FO probe, where surface plasmons were resonantly excited at the gold-coated FO tip. The back-reflected light was collected in a spectrometer (HR2, OceanOptics, Inc.) connected to the second arm of the Y-optical splitter. The resulting spectrum was normalized to that of the tip in air.

The spectrometer was operated with an integration time of 4000 µs, and 20 consecutive spectra were averaged per recorded measurement point. A custom LabVIEW program was employed to monitor the temporal evolution of the plasmonic resonance by fitting and tracking the minimum of the reflectance dip over time.

### 2.3 Fabrication of organic electrochemical transistors (OECTs)

The OECTs were fabricated following a standard photolithography process. A Cr/Au bilayer (10/100 nm) was sputter-deposited onto clean glass substrates (26×76 mm) and patterned using conventional photolithography techniques, yielding the source and drain contacts and gate electrodes. The devices were then encapsulated with a two-layer parylene C coating: the first layer provided electrical insulation, while the second served as a sacrificial layer. Selective removal of parylene C from the active channel and the electrode areas was achieved by reactive ion etching (RIE) in an O₂ plasma atmosphere.

For the OECT channel, a 5% v/v ethylene glycol (to enhance conductivity), 1% v/v glycidyloxypropyltrimethoxysilane (GOPS, as a cross-linking agent), and 0.1% v/v dodecylbenzenesulfonic acid (DBSA, to promote uniform film formation) were added to a commercial suspension of PEDOT:PSS (Clevios PH1000). The suspension was spin-coated onto the patterned devices at 3000 rpm for 60 s. Following deposition, the devices were baked at 90 °C for 1 min, and then the sacrificial parylene C layer was removed together with excess PEDOT:PSS, to define the channel geometry. Afterward, an additional thermal annealing step was performed at 140 °C for 1 h to complete solvent evaporation and ensure film stability. The OECTs were then soaked in Milli-Q water overnight to remove residual low-molecular-weight molecules from the channel.

All OECTs used in this study featured the following geometry: channel width (W) = 1 mm, channel length (L) = 0.3 mm, film thickness (t) ≈ 40-60 nm, and gate electrode diameter = 4 mm. The channel thicknesses were measured using profilometry (Bruker, DektakXT Stylus Profiler). A scratch was made on the film using a needle, and the resulting step height was measured to determine the film thickness (Supporting Information Figure S17). Measurements were taken at four different positions per device (Supporting Information Table S2).

### 2.4 OECT electrical measurements

Electrical measurements of the OECTs were conducted using a Keithley 2604B source meter controlled via SweepMe! software. Output curves (*I_D_* vs. *V_DS_*) were obtained by sweeping *V_DS_* from 0.1 V to −0.5 V at gate-source voltages (*V_GS_*) incremented from -0.1 to 0.5 V in 0.1 V steps. Transfer characteristics (*I_D_* vs *V_GS_*) were recorded by sweeping *V_GS_* from −0.2 to 0.6 V at drain-source voltages (*V_DS_*) stepped from −0.2 to −0.4 V in −0.2 V increments. Before aptamer functionalization and sensing experiments, freshly fabricated OECTs were precycled by repeatedly recording output and transfer curves until the electrical responses stabilized. Stability was confirmed by comparing the final transfer curve before and after gate electrode incubation in pure PBS buffer.

## 3. Characterization Methods

### 3.1 Atomic force microscopy (AFM)：

The morphology and thickness of the pALT films were characterized using AFM (Bruker Dimension ICON) operated in tapping mode with a cantilever resonance frequency of 285 kHz and a normal spring constant of 42 N·m^-1^. AFM images were processed using Gwyddion 2.63 SPM data visualization and analysis software. To determine the thickness of pALT nanofilms, a mechanical scratch was introduced to expose the underlying substrate, and the height difference between the scratched gold substrate and the pALT nanofilm surface was measured using a one-dimensional statistical function. Surface roughness was quantified based on statistical parameters extracted from the image data.

### 3.2 Scanning electron microscopy (SEM):

High-resolution images of the pALT films were obtained using a LEO Gemini 1530 scanning electron microscope. Samples were mounted onto aluminium SEM stubs using high-purity carbon adhesive tabs. Imaging was performed at an accelerating voltage of 3 kV with a working distance of 1.4 mm.

### 3.3 X-ray photoelectron spectroscopy (XPS):

XPS was conducted using a Kratos Axis UltraDLD spectrometer 3 (Kratos, Manchester, England) using an Al K excitation source with a photon energy of 1487 eV. Processing of the spectra was accomplished by CasaXPS Release 2.3.16 software. For the analysis of high-resolution spectra, all peaks were modelled using a Shirley background and charge-referenced to the C–C/C–Hx peak at 284.8 eV. Peak shapes were modelled with mixed Gaussian–Lorentzian line shapes (70% Gaussian/30% Lorentzian, GL(30)).

### 3.4 Fourier transform infrared spectroscopy (FT-IR):

FT-IR measurements were performed using infrared reflection absorption spectroscopy under controlled humidity in the sample chamber. Spectra were recorded with 1000 scans at a resolution of 4 cm^-1^. Samples were mounted on a translational stage, allowing alternating reflections between the sample deposited on the gold substrate and a bare gold substrate. Reflections from the bare gold were used as background to compensate for water vapor absorption.

### 3.5 Kelvin probe measurements (KP):

The KP measurements were performed using an ambient single-point Kelvin probe system from Anfatec, with a probe tip diameter of 1.4 mm. The results are provided as average values from at least three samples, with each sample having at least two spot measurements.

### 3.6 Confocal microscopy measurements:

Confocal fluorescence imaging of aptamer-functionalized pALT5c on gold substrates was conducted using a custom-built confocal microscope. Samples were mounted in an inverted orientation and excited with a 532 nm laser, focused onto the specimen through an oil-immersion objective (Nikon PLAN 100x, N.A. = 1.35). The emitted fluorescence was collected through the same objective, filtered using a 575/40 nm band-pass filter, and detected with an avalanche photodiode (APD). Images were acquired over a 200 x 200 μm^2^ field of view with a step size of 0.0573 μm. Image processing and data visualization were performed using Origin 2024b.

### 3.7 Electrochemical impedance spectroscopy (EIS) of OECT:

EIS measurements on OECT were conducted using a potentiostat (PlamSens4) in a standard three-electrode configuration. An Ag/AgCl pellet electrode (Warner Instruments) served as the reference electrode, and a platinum electrode was used as the counter electrode. The working electrode was either the gold electrode or the PEDOT:PSS channel, depending on the experiment. Measurements were carried out under open-circuit potential (OCP) in PBS solution, pH 7.4, with an AC amplitude of 10 mV over a frequency range from 10 kHz to 0.5 Hz.

### 3.8 Strain-induced elastic buckling instability for mechanical measurements (SIEBIMM):

To determine Young’s modulus of the pALT nanofilms, films were first lifted from the gold electrode surface and transferred using a sacrificial polyvinyl alcohol (PVA) support layer as previously described^4^. Gold-coated substrates bearing pALT nanofilms were incubated in an aqueous EDC/NHS solution (200 mM/50 mM) for 2 h to further crosslink the film; films were then subjected to chronoamperometry at 1.2 V for 600 s. Then, a micrometer-thick sacrificial PVA layer (200 µL of 10 wt% aqueous PVA, drop-cast) was applied to the film surface and dried at 40 °C for ≈25 min. Subsequently, the PVA-coated pALT films were peeled from the gold substrate and transferred onto a pre-stretched PDMS substrate (~20% strain) using a custom compression-stretching apparatus. The PVA was removed by dissolution in Milli-Q water, and the samples were dried in the fume hood. Then, the pALT film-coated pre-stretched PDMS substrates were fixed again with screw clamps to the home-built apparatus. Then the applied strain was relaxed, producing the buckling of the pALT films due to compression. The wavelength λ of the wrinkles was determined by AFM (Bruker Dimension ICON) with a Si cantilever of 70 kHz resonance frequency and a force constant of 2 N m^-1^ in tapping mode. The Young’s modulus of the pALT nanofilm was calculated using Equation (1)^5^:

$$E_{f}=3E_{S}\frac{(1-v_{f}^{2})}{(1-v_{s}^{2})} \frac{\lambda}{2\pi t}$$

Where *E_f_* is the Young’s modulus of the pALT nanofilms, *λ* is the wavelength of the periodic wrinkles induced in the film upon buckling, *E_f_* is the Young’s modulus of the PDMS substrate, *v_s_* is the Poisson’s ratio of the substrate (assumed to be 0.5), *v_f_* is the Poisson’s ratio of the pALT film (assumed to be 0.33), and *t* is the film thickness.

### 3.9 Semi-quantitative estimation of hybridization-accessible aptamer coverage

The hybridization-accessible aptamer coverage was estimated using a solution-based fluorescence depletion assay with a Cy3-labelled complementary DNA strand. pALT films on gold substrates were functionalized with the amine-terminated aptamer as described in the main text. A fluorescence calibration curve was first established by measuring Cy3-labelled complementary strand solutions with known concentrations (0, 0.001, 0.01, 0.1, 1, and 10 μM) on a Tecan Spark 20M multimode microplate reader (Tecan, Switzerland), with excitation at 550 nm and emission at 570 nm, using a bandwidth of 5nm.

For the depletion assay, 30 μL of 10 μM Cy3-labelled complementary strand solution was incubated with aptamer-functionalized pALT surfaces with a defined exposed area of 28.26 mm^2^ for 30 mins. Three independently prepared samples were analysed. After incubation, the post-incubation solution was collected as Wash 0. The samples were then rinsed twice with 30 μL nuclease-free water for 10 min each. During each rinsing step, the rinsing solution was gently pipetted several times over the sample surface and then collected as Wash 1 and Wash 2, respectively. All collected fractions were quantified using the fluorescence calibration curve. The amount of complementary strand retained on the surface was estimated by subtracting the total recovered amount in Wash 0–2 from the initial input amount.

### 3.10 Low-magnification wide-field fluorescence imaging

Low-magnification wide-field fluorescence images were acquired to assess the macroscopic distribution of Cy3 signal on the aptamer-functionalized pALT surfaces. The samples were imaged through a ChemiDoc MP Imaging System. Gel-documentation visible-light images were acquired using the colorimetric mode, while the wide-field fluorescence images were acquired using the Cy3 mode.

### 3.11 Sensor stability tests after storage

The storage stability of aptamer-functionalized pALT sensing interfaces was evaluated using both FO-SPR and OECT platforms. The sensing measurements were performed following the same protocols as described for freshly prepared sensors.

For FO-SPR measurements, the aptamer-functionalized pALT-coated fibre-optic sensor was continuously immersed in PBS on the FO-SPR instrument, enabling real-time in situ monitoring of the sensing interface. IL-6 sensing measurements were performed after 3 or 7 days of continuous storage in PBS at room temperature.

For OECT measurements, aptamer-functionalized pALT gate electrodes were stored in PBS at 4 °C and tested after 3 or 7 days.

### 3.12 Redox-probe electrochemical impedance spectroscopy (EIS) and cyclic voltammetry (CV) on Au substrates

EIS and CV were performed to evaluate the interfacial charge-transfer properties of pALT- and SAM-based biointerfaces. Measurements were carried out in PBS (pH 7.4) containing a 5 mM ferri/ferrocyanide redox couple using a Metrohm Autolab PGSTAT204 potentiostat in a three-electrode configuration, with the modified Au substrate as the working electrode, an Ag/AgCl reference electrode, and a Pt wire counter electrode.

Carboxyl-terminated SAMs on gold substrates were formed by immersing cleaned Au electrodes in a 10 mM ethanolic solution of 11-MUA overnight. Bare Au, pALT-coated Au, 11-MUA SAM-modified Au, aptamer-functionalized pALT-coated Au, and aptamer-functionalized 11-MUA SAM-modified Au electrodes were measured under identical conditions. EIS spectra were collected at the open-circuit potential using a 10 mV sinusoidal perturbation over a frequency range of 10^5^ to 0.1 Hz. CV was recorded from a potential of -0.1 to 0.5 V with three cycles at a scan rate of 20 mV s^-1^.

# Supporting Text

## Discussion on the detection mechanism

The concentration-dependent shift of the transfer characteristics (Figure 5F) can be interpreted using the Bernard & Malliaras model, which describes the drain current of p-type depletion-mode OECTs according to Equations (2-6)^6^:

$I_{DS}=G\left[ 1-\frac{1}{V_{P}}\frac{C_{G}}{C_{ch}+C_{G}}\left( V_{GS}+V_{off}-\frac{V_{DS}}{2} \right) \right]V_{DS}$ (2)

$G=\frac{Wt}{L} q\mu\rho_{0}$ (3)

$V_{p}=\frac{q\rho_{0}}{C^{*}}$ (4)

$C_{G}=C_{G,A} A_{G}$ (5)

$C_{ch}=C^{*} WLt$ (6)

Where *G* represents the channel conductance, *Vp* is the pinch-off voltage, *C_G_* and *C_ch_* denote the capacitance of the gate and channel, respectively, *V_GS_* is the applied gate voltage, and *V_off_* is the voltage shift at the gate–electrolyte interface induced by biological binding events. *V_DS_* is the applied drain voltage. The channel geometry is defined by its width (*W)*, thickness (*t),* and length (*L).* Additionally, *q* is the elementary charge, *µ* is the mobility, *ρ_0_* is the initial charge density in the channel, *C_G,A_* is the area-normalized capacitance of the gate interface, *A_G_*, is the effective gate-electrode area, *C** is the volumetric capacitance of the channel, and *C_G_*/(*C_ch_*+*C_G_*) is referred to as the capacitive coupling factor.

To elucidate the origin of the transfer curve shift, we examined two key parameters in the Bernard & Malliaras model: the capacitive coupling factor and the offset voltage (*V_off_*). Electrochemical impedance spectroscopy (EIS; Figure S20), Kelvin probe measurements (KP; Figure S21), and open-circuit potential (OCP; Figure S22) measurements were performed to evaluate their respective contributions.

Qualitatively, these two effects are expected to influence the transfer characteristics in opposite directions. EIS measurements (Figure S20) showed that the gate capacitance increased by approximately 50% after incubation with 100 ng mL^-1^ IL-6. According to Equation (2), an increase in gate capacitance enhances the capacitive coupling between the gate and the channel, thereby increasing the effective gate voltage experienced by the channel. For depletion-mode PEDOT:PSS OECTs, this effect would shift the transfer characteristics toward lower applied gate voltages. In contrast, KP and OCP measurements revealed a pronounced IL-6-induced interfacial potential offset at the aptamer-functionalized gate. Specifically, the KP surface potential (V_SPD_) decreased by approximately 94.5 mV after IL-6 binding, whereas the OCP measured in PBS increased by approximately 120 mV (Supporting Fig. S21A and S22). Although these measurements are performed under different environments and follow different sign conventions, both support a substantial binding-induced potential change at the sensing gate. This offset is equivalent to introducing an additional negative gate-bias contribution, so that a higher applied gate voltage is required to maintain the same drain current. Therefore, the interfacial potential change shifts the transfer characteristics toward higher gate voltages, opposite to the shift expected from the capacitance increase.

Because the capacitive contribution would partially compensate the sensing signal arising from the interfacial potential variation, the device geometry was chosen to minimize its influence. According to Equations (5) and (6), the gate capacitance scales with the effective gate-electrode area, whereas the channel capacitance scales with the channel volume. In the present device, *C_ch_* =0.52 μF, *C_G_* of the aptamer-functionalized pALT gate is 1.92 μF, and *C_G_* of the aptamer-functionalized pALT gate after incubation with 100 ng mL^-1^ IL-6 is 2.83 μF. Thus, *C_G_* is approximately four to six times larger than *C_ch_*. Using these capacitance values, the coupling factor *C_G_*/(*C_G_*+*C_ch_*) increases only from 0.80 to 0.84 after IL-6 binding. At *V_GS_* = 0.2 V, this corresponds to an increase in the effective gate voltage from 0.160 to 0.168 V, that is, only about 8 mV. Consequently, the same channel modulation would be achieved at a slightly lower applied gate voltage, approximately 0.160/0.84=0.190 V instead of 0.200 V, corresponding to only a minor shift of the transfer characteristics toward lower gate voltages.

Although this quantitative estimate should be regarded as approximate, it shows that the capacitance-induced shift is much smaller than the measured interfacial potential variation and, importantly, occurs in the opposite direction to the experimentally observed transfer-curve shift. These observations support the conclusion that the OECT sensing response is primarily governed by the IL-6-binding-induced interfacial potential change at the aptamer-functionalized gate, rather than by changes in capacitive coupling.

# Supporting Figures

Figure S1. AFM height images of the pALT films on gold substrates with ten (A), fifteen (B), and twenty (C) cycles. AFM 3D height images of the pALT films with ten (D), fifteen (E), and twenty (F) cycles.

Figure S2. SEM images of the pALT films with ten (A, D), fifteen (B, E), and twenty (C, F) cycles.

Figure S3. Literature-reported electropolymerization motifs of structurally related phenolic/aminophenolic monomers used for comparison with 3-amino-ʟ-tyrosine. (A) The proposed phenoxazine ladder structure for the electropolymerization of o-aminophenol, as reported by Kunimura et al.^7^ (B) The mechanism diagram of the electropolymerization of ʟ-tyrosine, as reported by Chu et al.^8^ These structures are included only as mechanistic analogies to rationalize possible oxidation and coupling motifs in 3-amino-ʟ-tyrosine-derived pALT, and do not represent the monomer or exact product structure used in this work.

Figure S4. FT-IR spectrum of 3-amino-ʟ-tyrosine. The broad feature around 3340 cm⁻¹ is attributed to overlapping O–H and N–H stretching vibrations. The band at 1745 cm⁻¹ is assigned to the carboxylic C=O stretching vibration. The band at 1637 cm⁻¹ is assigned mainly to N–H deformation of amino groups, with possible contribution from aromatic ring vibrations. The band at 1583 cm⁻¹ is attributed to aromatic C=C stretching, while the feature at 1353 cm⁻¹ is assigned to phenolic O–H in-plane bending^9, 10^.

## **Figure S5.** (A) XPS spectrum of O, N, and C of pALT5c film. (B) Deconvolution of the N1s XPS peak of pALT5c film. (C) Deconvolution of the O1s XPS peak of pALT5c film.

Figure S6. (A) XPS survey spectrum of 11-mercaptoundecanoic acid (11-MUA). (B) Deconvolution of the C1s XPS peak of 11-MUA. Carboxyl-terminated SAMs on gold substrates were formed by immersing cleaned Au electrodes in a 10 mM ethanolic solution of 11-MUA overnight.

Figure S7. XPS characterization of pALT films after 10 days of incubation in different media. Survey spectra and high-resolution C1s spectra are shown for pALT films incubated in DMSO (A,B), hexane (C,D), pH 2 Milli-Q water (E,F), pH 10 Milli-Q water (G,H), and PBS (I,J). The C1s spectra were deconvoluted into C–C/C–Hx, C–O/C–N, C=O/C=N, and O–C=O components using a Shirley background. Increased Au4f contributions under DMSO, hexane, and pH 2 conditions, together with largely unchanged C1s component distributions and intact SEM morphology (Figure 2A), suggest limited interfacial reorganization and/or partial removal of weakly bound oxygen-rich oligomeric species rather than extensive film degradation. The sample after pH 10 Milli-Q water incubation showed increased C–C/C–Hx and decreased C–O/C–N contributions, indicating alkaline-induced reorganization or further coupling/crosslinking of residual oligomeric species.

Figure S8. X-Y axis confocal microscopy images for pALT film on Au substrate with the following ssDNA incubation steps: (A) Functionalization with Apt only and (B) Apt’ only. Where Apt = Aptamer, Apt’ = Cy3-labeled complementary sequence.

Figure S9. Low-magnification images of aptamer-functionalized pALT-coated Au samples after hybridization with the Cy3-labelled complementary strand and the corresponding controls. Gel-documentation visible-light images were acquired using the colorimetric mode of the gel documentation system. Wide-field fluorescence images were acquired using the fluorescence mode (Cy3), and macroscopic photographs show the overall appearance of the samples. Apt is the aptamer, Apt’ is the Cy3-labelled complementary strand, while Scr is the Cy3-labelled scrambled control sequence.

Figure S10. Semi-quantitative estimation of hybridization-accessible aptamer coverage on the pALT surface using a Cy3-labelled complementary strand depletion assay. A known amount of Cy3-labelled complementary strand (10 uM, 30 uL) was incubated with the aptamer-functionalized pALT-coated Au surface with a defined area of 28.26 mm^2^. After incubation, the post-incubation solution (Wash 0) and two washing fractions (Wash 1 and Wash 2) were collected and quantified using a solution fluorescence calibration curve. The amount of surface-retained complementary strand was estimated by subtracting the total recovered amount in Wash 0–2 from the initial input amount. This analysis gave an estimated retained amount of 76.08 ± 28.57 pmol, corresponding to an approximate hybridization-accessible aptamer coverage of 2.69 ± 1.01 pmol mm^-2^. Error bars represent standard deviations. For the OECT gate electrode with a diameter of 4 mm, this coverage corresponds to an estimated hybridization-accessible aptamer amount of ~33.8 pmol. Assuming 1:1 binding between IL-6 and the immobilized aptamer, this value represents an upper-bound estimate of the IL-6 binding capacity of the gate surface. By comparison, the highest IL-6 concentration used in the OECT experiment, 1 μg mL^-1^ in 50 μL, corresponds to approximately 2.4 pmol IL-6 based on the molecular weight (20.9 kDa) of the IL-6 used in this study. Therefore, the estimated amount of hybridization-accessible aptamer on the gate should be substantially enough for binding with the total amount of IL-6 introduced, supporting sufficient receptor availability for the OECT sensing measurements.

Figure S11. FT-IR measurement. FT-IR spectra for pALT5c nanofilm and aptamer-functionalized pALT5c nanofilm.

Figure S12. SEM measurement. SEM image of pALT5c after aptamer functionalization. The nanoparticle-like surface features were not intentionally introduced as nanoparticles, but are attributed to localized organic domains formed during the multistep biofunctionalization and drying process.

Figure S13. The time-resolved response during the electropolymerization of pALT5c film, where changes in resonance wavelength and current were recorded simultaneously over time. Each forward scan shows an anodic current peak accompanied by a red-shift in resonance wavelength, consistent with monomer oxidation and film deposition at the interface. The partial blue-shift during the reverse scan is attributed to partial reduction and interfacial relaxation. The cumulative upward drift of the SPR baseline over successive cycles indicates net irreversible film growth.

Figure S14. (A) Wavelength shift of the resonance FO tip for the pALT5c film-based aptasensor in response to varying IL-6 concentrations in phosphate-buffered saline (PBS), as recorded by SPR (raw data). Any red-shift or blue-shift of wavelengths in the subsequent medium changes can be attributed to the signal shifting to the respective new baseline during the addition of IL-6 cytokines. (B) Independent repeat measurements obtained from separate sensors, showing reproducible IL-6 responses.

Figure S15. **(**A) Shift in wavelength of the resonance FO tip for the pALT5c electropolymerization and functionalization processes. (B) Shift in wavelength of the resonance FO tip with aptamer-functionalized pALT5c nanofilm, upon exposure to varying concentrations of HSA and then 100 ng mL^-1^ IL-6 in phosphate-buffered saline (PBS), as recorded by SPR (raw data). Any red-shift or blue-shift of wavelengths in the subsequent medium changes can be attributed to the signal shifting to the respective new baseline during the addition of proteins.

Figure S16. **(**A) Shift in wavelength of the resonance FO tip for the pALT5c electropolymerization and functionalization processes using EDC/NHS linkers but without aptamers. (B) Shift in wavelength of the resonance FO tip with the pALT5c film using the same fabrication process and functionalized using EDC/NHS linkers but without aptamers, upon exposure to varying concentrations of IL-6 in PBS, as recorded by SPR (raw data).

## **Figure S17.** (A) Microscopy image of an OECT channel with a scratch used for profilometry measurements. (B) The profilometry profile obtained from one sample. The height difference indicates the channel thickness, while the bump is due to material accumulation after the scratch was made.

Figure S18. (A) Output curves of a pristine OECT by sweeping *V_DS_* from 0.1 V to –0.5 V at *V_GS_* values between –0.1 V and 0.5 V. (B) Transfer curve and transconductance for *V_DS_* = −0.4 V. (C) Output curves of a pristine OECT by sweeping *V_DS_* from 0.1 V to –0.5 V at *V_GS_* values between –0.2 V and 0.6 V using another device. (D) Transfer curve and transconductance for *V_DS_* = −0.4 V using another device.

Figure S19. CV profiles for a pALT5c electropolymerization procedure on the gate electrode of an OECT.

Figure S20. Capacitance of the OECT measured by EIS in PBS buffer. (A) Capacitance of bare gold gate electrodes. (B) Capacitance of PEDOT:PSS channel. (C) Capacitance of the aptamer-functionalized pALT-coated gate electrode after incubation with increasing IL-6 concentrations. The data sets reveal a concentration-dependent increase in gate capacitance, indicating changes in the electrical double layer upon IL-6 binding at the aptamer-functionalized pALT-coated gate surface. (D) Summary of the capacitances of the PEDOT:PSS channel, bare gold gate electrodes, pALT-coated gate electrode functionalized with aptamers, and aptamer-functionalized pALT-coated gate electrode after incubation in 100 ng mL^-1^ IL-6. The capacitance values were obtained by fitting the EIS data using an equivalent circuit Rs-CPE. Rs represents the electrolyte resistance, and CPE is the non-ideal capacitor representing the bare gold electrode, the PEDOT:PSS channel, the aptamer-functionalized pALT-coated gate electrode, or the aptamer-functionalized pALT-coated gold gate electrode after exposure to IL-6. (E) Electrochemical impedance spectroscopy (EIS) Bode plot of the aptamer-functionalized pALT-coated gate electrode after exposure to 100 ng mL^-1^ IL-6 in PBS. The impedance magnitude and phase response indicate a predominantly non-faradaic capacitive behavior, with a high-frequency transition dominated by the solution resistance. This behavior supports the use of an Rs-CPE equivalent circuit to describe the overall capacitive response of the heterogeneous sensing interface.

Figure S21. Surface potential of aptamer-functionalized pALT5c films before and after cytokine incubation measured by Kelvin probe. Kelvin probe was performed under ambient conditions, and these data were used as qualitative support for IL-6-induced surface-potential changes. (A) The surface potential of pALT5c films electropolymerized on gold substrates and functionalized with aptamers before and after incubation with IL-6 (100 ng mL^-1^). Before IL-6 incubation, the surface potential was 247.8±16.0 mV, which decreased to 153.3±25.6 mV after incubation, indicating charge redistribution at the film surface due to the specific aptamer–IL-6 binding interactions. (B) The surface potential of pALT5c films electropolymerized on gold substrates and functionalized with aptamers before and after incubation with TNF-α cytokines (100 ng mL^-1^). Before TNF-α incubation, the surface potential was 302.4±16.3 mV, which remained constant at 311.3±10.3 mV after incubation.

Figure S22. Open-circuit potential measurements of the aptamer-functionalized pALT gate electrode in PBS solution. Error bars represent standard deviations. *V_OCP_* measured before (in PBS solution) and after IL-6 exposure (100 ng mL^-1^ IL-6). Before IL-6 exposure, the *V_OCP_* was 61.1±17.0 mV, which increased to 180.4±12.1 mV after incubation.

Figure S23. Analytical curve in log scale showing a linear behavior. The dashed line is the linear fit used to calculate the LOD. The red dot represents the blank test, in which the OECT-based aptasensors were incubated in pure PBS to assess the device stability. The value corresponds to the average response of five devices, and the error bar (not visible as the standard deviation is much smaller than the dot) was calculated using the standard deviation.

Figure S24. Sensing stability of aptamer-functionalized pALT sensors. (A,B) FO-SPR responses of aptamer-functionalized pALT-coated Au fibre sensors to IL-6 after 3 days (A) and 7 days (B) of storage in PBS. Concentration-dependent resonance wavelength shifts were retained after storage, although the response at higher IL-6 concentrations became compressed compared with freshly prepared sensors. (C) OECT responses of aptamer-functionalized pALT gate electrodes after storage at 4 °C. The gate-voltage shift, *ΔV_GS_*, decreased after 3 and 7 days compared with fresh devices, but remained above the noise level and retained a concentration-dependent trend, indicating preserved IL-6 sensing capability with partially reduced interfacial transduction efficiency.

Figure S25. Comparison of interfacial charge-transfer properties of pALT5c- and 11-MUA SAM-modified Au electrodes. Carboxyl-terminated SAMs on gold substrates were formed by immersing cleaned Au electrodes in a 10 mM ethanolic solution of 11-MUA overnight. EIS (A, C) and Cyclic voltammetry (B,D) were recorded in 5 mM [Fe(CN)_6_]^3−/4−^ in PBS. pALT5c-coated Au retained clear ferri/ferrocyanide redox peaks and showed a lower charge-transfer resistance than the carboxyl-terminated 11-MUA SAM. After aptamer immobilization, the pALT5c-based interface continued to exhibit lower interfacial resistance than the aptamer-functionalized SAM, indicating that pALT forms a less charge-transfer-blocking biointerface while enabling covalent aptamer coupling.

# Supporting Tables

Table S1. Elemental composition of the pALT5c film determined by XPS. Carbon, Nitrogen, and Oxygen quantification and components.

| **Name** | **Position** | **Raw area** | **At Conc [%]** | **Conc [%]** | **Functional groups** |
| --- | --- | --- | --- | --- | --- |
| O1sA | 531.58 | 5845.0 | 9.93 | 39.87 | C=O |
| O1sB | 532.66 | 8147.7 | 13.85 | 55.61 | C–O |
| O1sC | 535.20 | 660.1 | 1.12 | 4.51 | –OH shake-up |
| **O** |  |  | **24.90** | **100.00** |  |
| N1sA | 398.96 | 1334.7 | 3.08 | 29.41 | =NR |
| N1sB | 399.91 | 2289.1 | 5.28 | 50.49 | –R_2_NH |
| N1sC | 401.36 | 909.3 | 2.10 | 20.10 | –RNH_2_ |
| **N** |  |  | **10.46** | **100.00** |  |
| C1sA | 284.80 | 7442.1 | 31.37 | 48.53 | C–C, C–H_x_ |
| C1SB | 286.25 | 4421.8 | 18.67 | 28.89 | C–O, C–N |
| C1sC | 287.76 | 1891.8 | 8.00 | 12.38 | C=O, C=N |
| C1sD | 288.75 | 1557.5 | 6.60 | 10.80 | O–C=O |
| **C** |  |  | **64.64** | **100.00** |  |

Table S2. Thickness of the PEDOT:PSS layer.

| **Devices** | **Thickness (nm)** | | | | **Average (nm)** | **Standard deviation (nm)** |
| --- | --- | --- | --- | --- | --- | --- |
| **1** | 33 | 32 | 39 | 38 | 36 | 4 |
| **2** | 40 | 41 | 42 | 40 | 41 | 1 |
| **3** | 42 | 45 | 40 | 39 | 42 | 3 |
| **4** | 66 | 64 | 57 | 77 | 66 | 8 |
| **5** | 48 | 43 | 44 | 51 | 47 | 4 |
| **6** | 56 | 53 | 55 | 48 | 53 | 4 |
| **7** | 51 | 50 | 52 | 57 | 52 | 3 |
| **8** | 67 | 63 | 59 | 49 | 59 | 8 |
| **9** | 50 | 66 | 54 | 66 | 59 | 8 |
| **10** | 59 | 55 | 57 | 52 | 56 | 3 |

Table S3. Comparison of biosensors for detecting IL-6, listing the technique, receptor type used, the tested lower limit, and the dynamic detection range.

| **Technique** | **Receptor type** | **Tested lower concentration limit (g mL^-1^)** | **Detection range** | **Reference** |
| --- | --- | --- | --- | --- |
| Screen printed OECTs | Aptamer | 2.09E-9 | 2.09E-9～2.09E-8 | ^11^ |
| Planar OECT | Aptamer | 2.09E-10 | 2.09E-10～2.09E-6 | ^12^ |
| OECT | Antibody | 2.0E-9 | 2.0E-9～2.2E-6 | ^13^ |
| OECT | Antibody | 2.09E-11 | 2.09E-11～2.09E-7 | ^14^ |
| Au-functionalized wrinkle graphene field-effect transistor (FET) | Antibody | 6.48E-13 | 6.48E-13～6.48E-7 | ^15^ |
| Graphene-based fully integrated portable nanosensing system | Aptamer | 1.05E-9 | 1.05E-9～1.7E-8 | ^16^ |
| Cationic polyelectrolyte encapsulated black phosphorene-based impedimetric immunosensor | Antibody | 3E-12 | 3E-12～7.5E-8 | ^17^ |
| Graphene FET | Aptamer | 1.05E-9 | 1.05E-9～5.05E-8 | ^18^ |
| Surface-enhanced Raman spectroscopy | Antibody | 5E-13 | 5E-13～5E-8 | ^19^ |
| Surface-enhanced Raman spectroscopy | Aptamer | 2.09E-11 | 2.09E-11～2.09E-6 | ^20^ |
| Surface plasmon-enhanced fluorescence spectroscopy | Antibody | 2E-12 | 2E-12～2E-9 | ^21^ |
| **This work** | **Aptamer** | **1E-13** | **1E-13～1E-8** |  |

# References

1.	Marchesi D'Alvise, T., Sunder, S., Hasler, R. et al. Preparation of ultrathin and degradable polymeric films by electropolymerization of 3-amino-l-tyrosine. *Macromolecular Rapid Communications* 44, 2200332 (2023).

2.	Yu, J., D’Alvise, T.M., Harley, I. et al. Ion and molecular sieving with ultrathin polydopamine nanomembranes. *Advanced Materials* 36, 2401137 (2024).

3.	Pollet, J., Delport, F., Janssen, K.P.F. et al. Fiber optic SPR biosensing of DNA hybridization and DNA–protein interactions. *Biosensors and Bioelectronics* 25, 864-869 (2009).

4.	Marchesi D'Alvise, T., Harvey, S., Hueske, L. et al. Ultrathin Polydopamine Films with Phospholipid Nanodiscs Containing a Glycophorin A Domain. *Advanced Functional Materials* 30, 2000378 (2020).

5.	Stafford, C., Harrison, C., Beers, K. et al. A buckling-based metrology for measuring the elastic moduli of polymeric thin films. *Nature Materials* 3, 545-550 (2004).

6.	Torricelli, F., Adrahtas, D.Z., Bao, Z. et al. Electrolyte-gated transistors for enhanced performance bioelectronics. *Nature Reviews Methods Primers* 1, 66 (2021).

7.	Kunimura, S., Ohsaka, T. & Oyama, N. Preparation of thin polymeric films on electrode surfaces by electropolymerization of o-aminophenol. *Macromolecules* 21, 894-900 (1988).

8.	Chu, Z. et al. In-situ electro-polymerization of l-tyrosine enables ultrafast, long cycle life for lithium metal battery. *Chinese Chemical Letters* 34, 107563 (2023).

9.	Natarajan, S., Shanmugam, G. & Martin Britto Dhas, S.A. Growth and characterization of a new semi organic NLO material: L-tyrosine hydrochloride. *Crystal Research and Technology* 43, 561-564 (2008).

10.	Ramaekers, R., Pajak, J., Rospenk, M. & Maes, G. Matrix-isolation FT-IR spectroscopic study and theoretical DFT(B3LYP)/6–31++G** calculations of the vibrational and conformational properties of tyrosine. *Spectrochimica Acta Part A: Molecular and Biomolecular Spectroscopy* 61, 1347-1356 (2005).

11.	Burtscher, B., Diacci, C., Makhinia, A. et al. Functionalization of PEDOT:PSS for aptamer-based sensing of IL6 using organic electrochemical transistors. *npj Biosensing* 1, 7 (2024).

12.	Diacci, C., Burtscher, B., Berto, M. et al. Organic Electrochemical Transistor Aptasensor for Interleukin-6 Detection. *ACS Applied Materials & Interfaces* 16, 61467-61474 (2024).

13.	Gentili, D., D’Angelo, P., Militano, F. et al. Integration of organic electrochemical transistors and immuno-affinity membranes for label-free detection of interleukin-6 in the physiological concentration range through antibody–antigen recognition. *Journal of Materials Chemistry B* 6, 5400-5406 (2018).

14.	Manco Urbina, P.A., Berto, M., Greco, P. et al. Physical insights from the Frumkin isotherm applied to electrolyte gated organic transistors as protein biosensors. *Journal of Materials Chemistry C* 9, 10965-10974 (2021).

15.	Dou, C., Wu, Z., Chen, W. et al. Au-functionalized wrinkle graphene biosensor for ultrasensitive detection of Interleukin-6. *Carbon* 216, 118556 (2024).

16.	Hao, Z., Pan, Y., Shao, W. et al. Graphene-based fully integrated portable nanosensing system for on-line detection of cytokine biomarkers in saliva. *Biosensors and Bioelectronics* 134, 16-23 (2019).

17.	Chandra Barman, S., Sharifuzzaman, M., Zahed, M.A. et al. A highly selective and stable cationic polyelectrolyte encapsulated black phosphorene based impedimetric immunosensor for Interleukin-6 biomarker detection. *Biosensors and Bioelectronics* 186, 113287 (2021).

18.	Hao, Z., Luo, Y., Huang, C. et al. An intelligent graphene-based biosensing device for cytokine storm syndrome biomarkers detection in human biofluids. *Small* 17, 2101508 (2021).

19.	Zhou, T., Lu, D., She, Q. et al. Hypersensitive detection of IL-6 on SERS substrate calibrated by dual model. *Sensors and Actuators B: Chemical* 336, 129597 (2021).

20.	Muhammad, M., Shao, C.-S. & Huang, Q. Aptamer-functionalized Au nanoparticles array as the effective SERS biosensor for label-free detection of interleukin-6 in serum. *Sensors and Actuators B: Chemical* 334, 129607 (2021).

21.	Toma, M. & Tawa, K. Polydopamine thin films as protein linker layer for sensitive detection of interleukin-6 by surface plasmon enhanced fluorescence spectroscopy. *ACS Applied Materials & Interfaces* 8, 22032-22038 (2016).
